# Climate change over the Mediterranean and current destruction of marine ecosystem

**DOI:** 10.1038/s41598-019-55303-7

**Published:** 2019-12-11

**Authors:** Go-Un Kim, Kyong-Hwan Seo, Deliang Chen

**Affiliations:** 1 Department of Atmospheric Sciences, Division of Earth Environmental System, Pusan National University, Busan, South Korea; 2 Research Center for Climate Sciences, Pusan National University, Busan, South Korea; 3 Department of Earth Sciences, University of Gothenburg, Gothenburg, Sweden

**Keywords:** Climate sciences, Ocean sciences

## Abstract

The Mediterranean is one of the most vulnerable regions to climate change and its summer climate is known to be affected by the South Asian summer monsoon (SASM) through the monsoon–desert teleconnection. In future, rainfall is expected to increase not only over the SASM area but also over the East Asian summer monsoon (EASM) and equatorial Atlantic regions. Here we show that the remote forcing regions affect the Mediterranean climate in the future. A subset of CMIP5 climate simulations exhibits an increase in the descending motion over the Western Mediterranean in the future. This strengthened subsidence comes from the SASM, EASM, and Atlantic forcings: the SASM and EASM heating induces the Gill-type Rossby wave response, and the Atlantic forcing causes the northeastward wave energy propagation. The sea surface temperature change over the Western Mediterranean is consistent with the subsidence change both in the future and in the recent decades. The chlorophyll-a concentration and fisheries landings have decreased in the recent period along with sea surface temperature warming. Our results suggest that special attention is required to conserve the marine ecosystem in the Mediterranean as climate warms.

## Introduction

The Mediterranean is one of the most prominent and vulnerable climate change “hotspots^1^” and responds quickly to atmospheric forcings^2^. The region has been known to affect human and natural systems^1–4^ including water management, human health, plant/marine diversity, agriculture, and socio-economic productivity. Furthermore, the Euro–Mediterranean region frequently experienced extreme climate and weather events such as the hottest summer 2003^4,5^ and 2010^6^. The magnitude and frequency of the extreme temperature events over land and sea tend to increase in recent years^3,4,7^ and are expected to increase in the future^3,8–10^.

Summer climate over the Mediterranean is characterized by hot/warm and dry conditions with a descending motion^11^. This characteristic climate has been related to several atmospheric and oceanic variations including the South Asian summer monsoon (SASM) circulation^12–15^, the West African monsoon/Sahel rainfall^11,16–18^, the summer North Atlantic Oscillation^19,20^, and the Atlantic Multidecadal Oscillation^20^. In particular, the large-scale atmospheric circulation in South Asia is revealed to mostly influence the summer climate and weather of the Mediterranean via atmospheric teleconnection. This teleconnection is known as the monsoon–desert mechanism^12^, in which remote diabatic heating formed by the SASM rainfall generates the descending motion over the Mediterranean and nearby region through a westward-propagating Rossby wave. Therefore, it is important to understand how the monsoon–desert process influences the Mediterranean summer weather and the atmospheric circulation in the Mediterranean region under global warming. A more detailed understanding would improve the seasonal predictability skill and climate simulation for the Mediterranean and Europe in the boreal summer^21^, and contribute to reducing damages caused by meteorological disasters in the future.

Changes in the precipitation over South Asia and the Mediterranean have been extensively studied^3,22–26^. The future SASM rainfall is anticipated to remarkably increase^3,22^, whereas the precipitation over most of the Euro–Mediterranean region is expected to decrease^3,22–26^. This opposite tendency^22^ is suggested to occur due to the monsoon–desert mechanism as mentioned above. Note that future precipitation associated with the East Asian summer monsoon (EASM)^3,22,27–29^, West African monsoon^3,22^, and Atlantic Intertropical Convergence Zone^3,22^ are also anticipated to increase and whether these change may cause an additional change of the Mediterranean climate has not been addressed.

In fact, future change in the circulation over the Mediterranean exhibits a rather inhomogeneous spatial pattern. Previous study has proposed that the descending motion over the Western Mediterranean (WM, 0–20° E, 25°–45° N) is expected to strengthen, whereas that over the Eastern Mediterranean (EM) is anticipated to weaken in the future^30^. The descending motion changes over the Mediterranean has been attributed to the local circulation change as well as monsoon induced heating^30^. The latter is the main source according to the monsoon–desert theory^12^. However, except for the SASM-related forcing, there exist various forcing mechanisms such as the EASM, West African monsoon, and Atlantic Intertropical Convergence Zone. Therefore, the whole picture of physical processes causing the circulation changes over the Mediterranean needs to be investigated.

### Future changes in the Mediterranean atmospheric circulation

**Figure 1:**
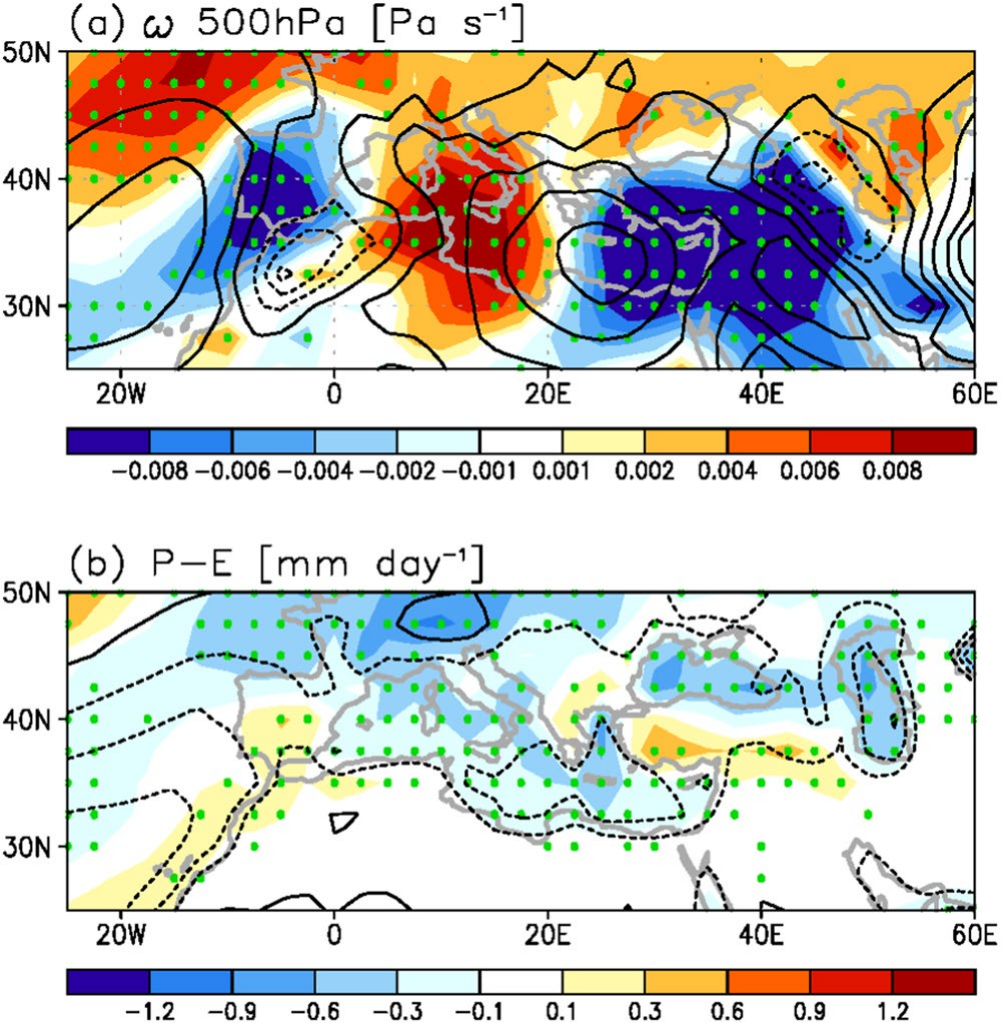
Multi-model ensemble-mean descending motion and P-E for climatology and future change during summer. (**a**) Spatial pattern of 500-hPa omega climatology (black contour; Pa s^−1^) and future change (shading; Pa s^−1^) for the seven-model ensemble-mean in boreal summer. (**b**) The same as in (**a**) but for the P-E (mm day^−1^). The contour intervals are 0.02 Pa s^−1^ in (**a**) and 1 mm day^−1^ in (**b**). Green dots in (**a,b**) indicate the points where more than 85% of the models agree on the sign of the mean for future change. In (**a,b**) maps were generated by GrADS version 1.9b4 (http://grads.iges.org/grads/).

The changes in the 500-hPa vertical wind (omega) and precipitation minus evaporation (P–E) during June–July–August (JJA) between the present climate (20 C) and future climate (21 C) are shown in Fig. 1. The climatological downward motion (corresponding to positive omega, black contour in Fig. 1a) is grossly located over 25°–45° N in the boreal summer. In particular, the descending motion over the EM (20°–40° E) is much stronger than that over other regions along 35° N. The climatological P–E value is negative across this subsidence region, implying a dry and warm climate, except in the Alps^24^ (Fig. 1b). On the other hand, future changes (shaded in Fig. 1a) demonstrate the positive omega anomalies over the WM (5°–20° E) but the negative omega anomalies over the EM. The result reveals that the descending motion would become stronger in the WM but with weaker descending motion in the EM in the future (Fig. 1a), which is in agreement with a previous study^30^. The circulation change tends to drive the gross feature of the P–E change; the drier atmosphere over the WM and the wetter one over the EM, although the latter P–E signal is weaker^24,25^. Furthermore, widespread downward motion anomalies are seen along 40°–50° N (Fig. 1a), indicating the poleward shift in the subtropical dry zone under warmer climate conditions^23,24,26,31^ and the resultant potential threat in water resources^24,31^.

### Remote heat sources inducing the atmospheric circulation change

**Figure 2:**
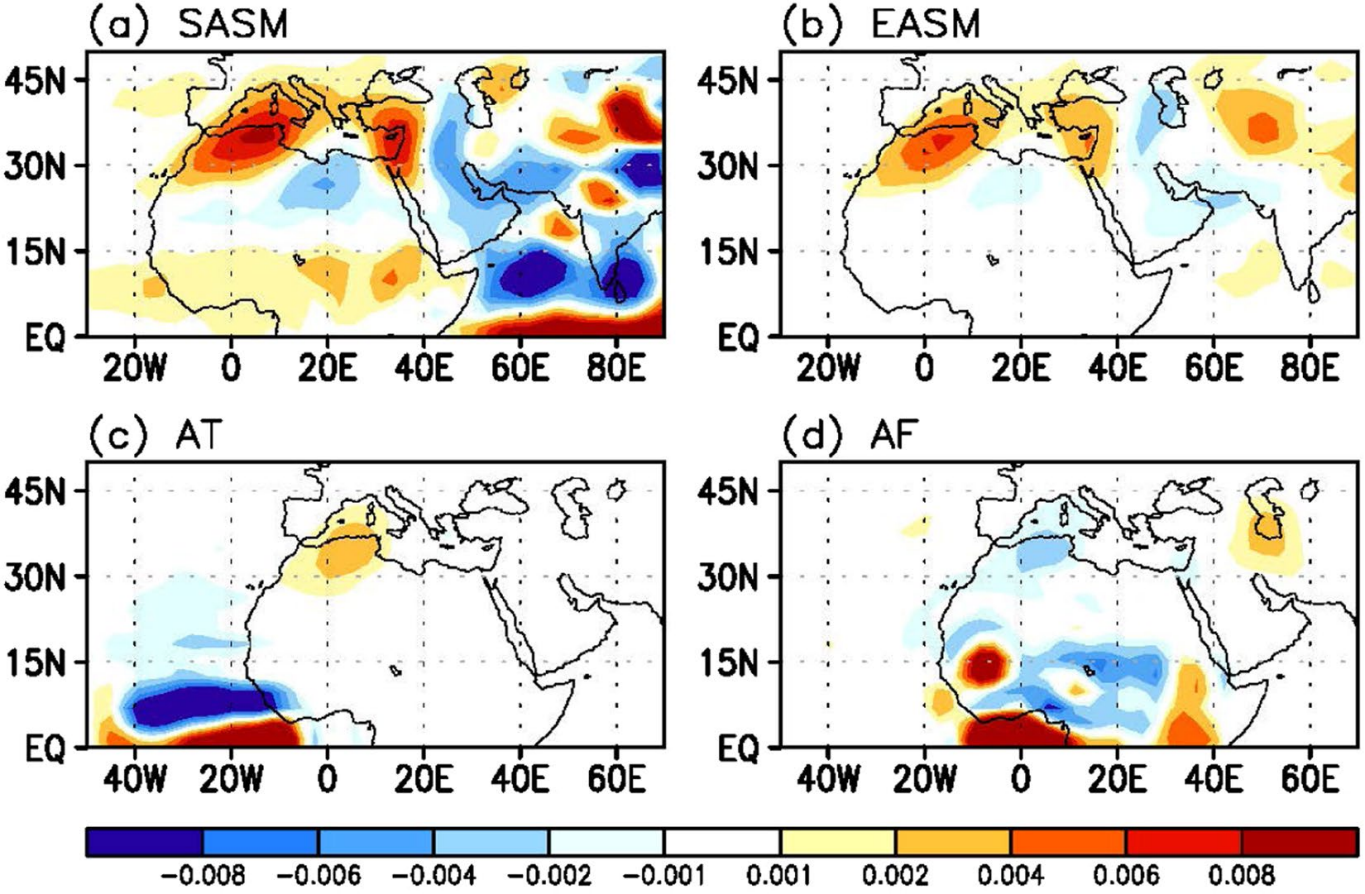
Simulated descending motion anomalies in response to each forcing. Middle troposphere (*σ* = 0.550) omega anomalies (shading; Pa s^−1^) averaged over 25–29 days of model simulation in response to the (**a**) SASM, (**b**) EASM, (**c**) Atlantic, and (**d**) African forcings. Maps were generated using GrADS version 1.9b4 (http://grads.iges.org/grads/).

The SASM has been considered a major remote forcing component inducing changes in the Mediterranean climate. However, this is not the only forcing source. To identify what additional heat sources are responsible for the atmospheric circulation change over the Mediterranean, the numerical experiments using Linear Balance Model (LBM) are performed. The input diabatic heating or cooling profiles are taken from the simulation outputs for the regions denoting the SASM, EASM, Atlantic, and Africa (indicated by the boxes in Supplementary Fig. S1b). The omega anomalies in the middle-troposphere (*σ* = 0.550) for the near steady state (which is achieved by the average over 25–29 day integration outputs) are shown in Fig. 2 for different heat sources. The results are not sensitive to the addition or exclusion of a few days after the 15-day integrations. The omega anomalies over 30°–40° N for the SASM forcing (Fig. 2a) show the generally similar zonal pattern to that of the 21 C CMIP5 omega changes (Fig. 1a) with positive anomaly over the WM and negative one over the nearby EM, although these two anomalies are shifted by 5° to the left. The EASM forcing (Fig. 2b) exhibits the similar dipole pattern with the SASM case (Fig. 2a), however with a weaker magnitude. This similarity comes from the use of the analogous heating profile between the two, which is top-heavy with strong heating in the upper troposphere and weak heating in the lower troposphere (blue and red lines in Supplementary Fig. S2).

The Atlantic source represents a portion of the Atlantic Intertropical Convergence Zone or it is considered as an Atlantic part of the whole West African monsoon rainfall. It is interesting to see that the Atlantic forcing (Fig. 2c) also induces the downward motion anomaly over the WM. Since the Atlantic forcing itself is weaker (Supplementary Fig. S2), the resulting response is also weaker compared to the SASM or EASM. On the other hand, the African source is the continental portion of the West African monsoon rainfall. This simulation is performed to check whether a local Hadley circulation affects the climate over the Mediterranean. The result (Fig. 2d) shows an ascending motion anomaly over the WM, which is an opposite circulation response compared to those of other experiments.

For a more quantitative attribution, the percentage ratio is estimated for the omega anomaly response over the WM (2.5°–20° E, 27.5°–45° N) for each experiment relative to those from total forcing (SASM + EASM + Atlantic). The descending anomalies of the SASM forcing shows a maximum ratio among the simulations, which amounts to ~53%. Those from the EASM and Atlantic forcings are 33% and 14%, respectively.

The magnitude and location of upward motion anomalies over the EM are not as well reproduced by all the experiments compared to the descending motion anomalies in the WM (Fig. 1a). This deficiency may come from the ignorance of the complex orography in the vicinity of the EM and Middle East basin^12,32,33^ (e.g., Taurus–Elburz–Zagros and Hindu Kush mountains).

### Possible mechanisms for the atmospheric circulation change

**Figure 3:**
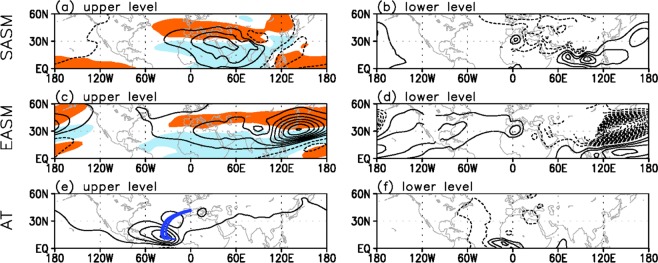
Simulated upper- and lower-level circulation anomalies in response to each forcing. Upper (σ = 0.230) and lower troposphere (σ = 0.950) streamfunction anomalies (contour; m^2^ s^−1^) averaged over 25–29 days for the (**a,b**) SASM, (**c,d**) EASM, and (**e,f**) Atlantic experiments. The intervals of streamfunction anomalies are in (**a,c**) 1.2 × 10^6^ m^2^ s^−1^, (**b,d**) 0.2 × 10^6^ m^2^ s^−1^, (**e**) 0.5 × 10^6^ m^2^ s^−1^, and (**f**) 0.15 × 10^6^ m^2^ s^−1^. Orange (sky blue) shading in (**a,c**) indicates the upper-level zonal wind anomaly greater (less) than 0.5 (–0.5) m s^−1^. Blue lines denote the Rossby wave ray path for waves with the zonal wavenumber 5. Maps were generated using GrADS version 1.9b4 (http://grads.iges.org/grads/).

To elucidate the physical processes of the Mediterranean circulation changes, the streamfunction anomalies for the SASM, EASM, and Atlantic forcing experiments are plotted (Fig. 3). The SASM and EASM experiments show that the anticyclonic circulation anomalies at the upper level expand westward into the Mediterranean from each of the forcing regions (Fig. 3a,c). This westward expansion is possible along the jet stream over the Eurasia region (10°–120° E, 30°–40° N) as shown in the Supplementary Fig. S3a. The jet stream area is associated with the large climatological meridional vorticity gradient which is proportional to the upper zonal wind (Supplementary Fig. S3a,c). It is likely that on the left (right) side of the local anticyclonic circulation anomaly, air with a lower (higher) planetary vorticity is advected from the south (north), enhancing (counteracting) the existing local vorticity^34^. Therefore, the streamfunction anomaly spreads to the west.

In the lower troposphere, in response to the positive heating associated with the Asian monsoon precipitation, cyclonic circulation anomalies are formed near the forcing regions (Fig. 3b,d), whereas anticyclonic circulation anomalies (albeit somewhat smaller) are developed over the northwestern part of Africa. The latter part of the circulation signal is barotropic if the upper-level circulation anomalies are considered together. By contrast, at the forcing region and its immediate western vicinity the baroclinic vertical structure is evident since the both forcings have the top-heavy diabatic heating profiles. The baroclinic spatial patterns are consistent with the structure of the monsoon–desert teleconnection pattern^12,13^, in which remote diabatic heating induces the low-level cyclonic circulation response to the west – then in combination with the upper-level anticyclonic circulation anomaly. This results in the development of warm temperature anomalies in the middle troposphere through the hydrostatic balance, stretching toward the Mediterranean. This thermal structure signifies the deepening of isentropic surface or the convex structure in this surface; then subsequently the descending motion is produced to the west of the curved isentropic surface along 35°–40° N, where the climatological winds are westerlies (orange shading in Fig. 3a,c); therefore, the adiabatic subsidence occurs over the WM.

Meanwhile, the upper-level streamfunction anomaly (contour in Fig. 3e) for the Atlantic forcing exhibits a wave-like pattern, propagating from the Atlantic to the Mediterranean. To verify the northeastward wave energy propagation, a ray-tracing technique is applied (see the Methods section for more details). The calculated arc-like rays for zonal wavenumber 5 (blue lines in Fig. 3e) trace well the circulation anomaly centers up to the Mediterranean, implying that the Atlantic forcing can induce the descending anomaly over the Mediterranean.

Then a question arises as to how the northeastward wave route can be formed even if a critical latitude (i.e., zonal wind is zero)^34^ exists near the equator as shown in Supplementary Fig. S3a. It is the climatological higher-level southerly wind (Supplementary Fig. S3b) that makes it possible for the wave energy to propagate to the north^35^. A complete Rossby wave theory that considers the inclusion of the meridional basic wind in the derivation of meridional group velocity leads to wave energy escaping from the easterly wind region.

### Impacts on the marine system over the mediterranean

The atmospheric circulation changes in the Mediterranean in the future could impact on the marine system there. Direct variables representing this marine environment change such as chlorophyll-a and fisheries are not available in the CMIP5 projection data. However, the sea surface temperature (SST) can be used for indirectly estimating the nutrient abundance and marine habitat condition in the future. The SST is expected to greatly increase over the WM than over the EM in the future (Supplementary Fig. S4b)^36^, although the climatological summer SST in the EM (Supplementary Fig. S4a) is higher. This SST change over the Mediterranean is consistent with the atmospheric circulation changes there (Fig. 1a).

**Figure 4:**
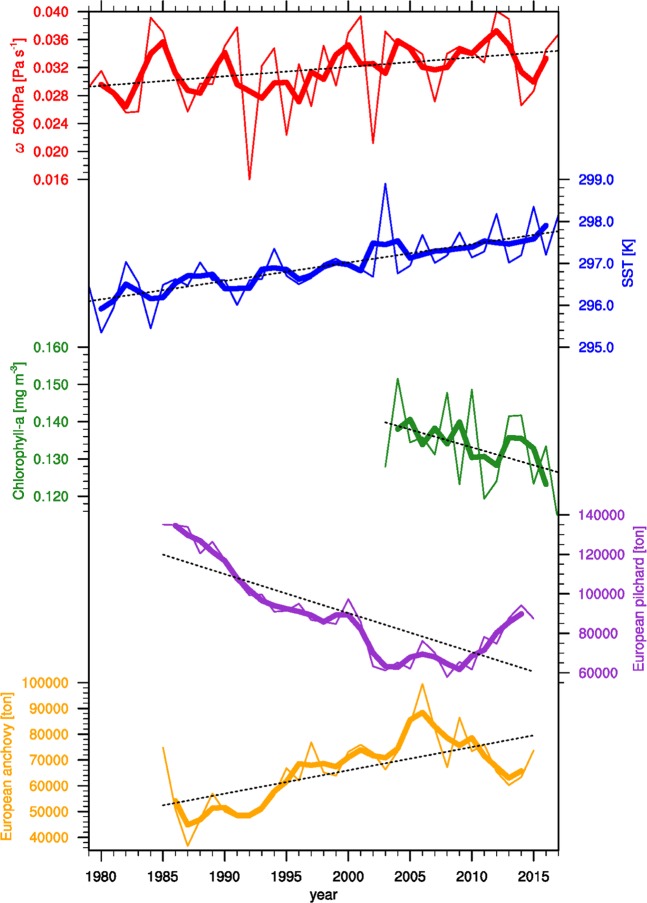
Observed global warming effects on the western Mediterranean. Time series of the summer-mean subsidence during 1979–2017 from the ERA-Interim (red line, Pa s^−1^, positive 500-hPa omega indicates the descending motion), the SST from the ERSSTv5 (blue line, K), the chlorophyll-a from the MODIS (green line, mg m^−3^), and the annual captured fisheries of European pilchard and anchovy from the FAO GMFC (purple and yellow lines, ton). Thin lines indicate the yearly value and thick lines denote the three-year running mean. Dotted black lines represent the best linear fit to time series of each variable.

However, still the effect of global warming on the marine ecosystem can be conjectured by examining the recent available data of chlorophyll-a and fisheries since the current warming effect is projected on the data. Over the recent decades (1979–2017), the descending motion in the WM has intensified 0.001 Pa s^−1^ per decade (red line in Fig. 4). The SST (blue line in Fig. 4) has also increased 0.4 K per decade in the WM (i.e., Adriatic, Balearic, Ligurian, and Tyrrhenian Sea)^37–39^. Their correlation in the three-year moving average is about 0.44, which is significant at the 99% confidence level. Also, the abundance of chlorophyll-a^40^, determined by MODIS data, has decreased since the early 2000s (green line in Fig. 4). The result suggests that this decreasing chlorophyll-a concentration can lead to shortage of food to marine organisms. The correlation coefficient for the WM SST with the chlorophyll-a is −0.71. The landings of European pilchard (purple line in Fig. 4) in the WM have decreased^41^ according to the fisheries landings dataset in the FAO-GFMC for the period 1985–2015, while those of the European anchovy have increase (yellow line in Fig. 4)^41^. Even though both the two fish species are caught most frequently during the warmer season, the spawning time for the European anchovy is from April to November, mostly during the warm season, whereas the European pilchard spawning occurs from September to May with peaks usually in the cold season^41,42^. The correlation of the fisheries landings for the European anchovy and pilchard with the SST is 0.67 and −0.78, respectively. When seawater warms, the oxygen would be less dissolved in the water and the more stratified vertical structure would develop; then nutrients cannot be supplied to the surface layer, decreasing the plankton concentration and fish populations^43^. Using other datasets (ERSSTv5, HadISSTv1.1, and NCEP R1 omega 500hPa) even for a longer period (1950−2017) produces nearly the same results (not shown).

The present results are consistent with many previous studies which showed that the decline in cold-water species (*Pseudocalanus elongatus*, *Clausocalanus spp*., and *Ctenocalanus vanus*)^39,44^ and in zooplankton *Penilia avirostris*^40^, and the increase in new species (*Diaixis pygmoea*)^39,44^ with warming SST since the late 1980s or early 1990s. In these studies, the fisheries landings data from seven Mediterranean European Union member states in 2008 have shown the decrease of 44% compared to the late 1990s^41^.

**Figure 5:**
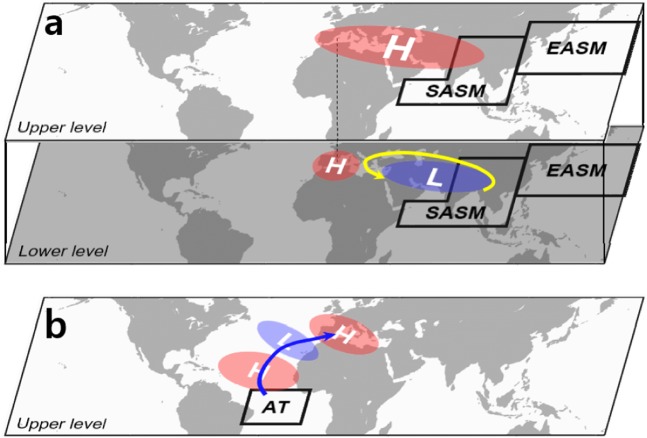
Remote control mechanisms for the heating over the western Mediterranean. Schematic illustrating the (**a**) monsoon–desert teleconnection and (**b**) northeastward wave energy propagation. Red (blue) shading ellipses indicate the anticyclonic (cyclonic) circulation. Black line boxes show the SASM, EASM, and Atlantic forcings. The Gill-type Rossby wave response to the SASM and EASM forcings is denoted by yellow line in (**a**) and the northeastward wave route emanated from the Atlantic forcing is shown as blue line in (**b**). The maps were created using the NCAR Command Language Version 6.4.0 (http://www.ncl.ucar.edu).

In summary, this study demonstrates the physical mechanisms (schematic illustration in Fig. 5) for changes in the descending motion over the Mediterranean under the global warming and potential impact of these changes on the marine ecosystem. The descending motion over the WM will become stronger in the future simulations, but weaker in the EM. This strengthened descending motion in the WM comes from not only the SASM forcing but also the EASM and the Atlantic forcings. Both the SASM and EASM forcings (Fig. 5a) induce the descending motion anomaly by Gill-type Rossby-wave response to the west of the remote forcing, as depicted in the monsoon–desert mechanisms^12^. The Atlantic forcing (Fig. 5b) generates the northeastward propagating wave train to the WM. The climatological upper-level southerly wind plays a critical role in the wave propagation across the critical latitude from the Atlantic source.

The pattern of the SST change in the Mediterranean in the future is consistent with that of the atmospheric circulation change. The WM (EM) SST is expected to greatly (lightly) increase along with the strengthening (weakening) in subsidence over the WM (EM). Interestingly, these characteristics also appear in the recent decades. In addition, chlorophyll-a concentration and total landing of fisheries over the WM have decreased during the recent period of the SST warming. This implies that change in the atmospheric circulation is one of the important factors in affecting the marine ecosystem over the Mediterranean. Recent studies have suggested that the serious water-column stratification^43^, marine heat waves^45^, and mortality of the marine ecosystem and fisheries industry^43,45,46^ can be exacerbated in the warmer climate. Therefore, the understanding of the atmospheric circulation change in the future provides insight into the marine system variation.

## Methods

### CMIP5 data

Datasets from a total of seven historical and RCP8.5 simulations from the World Climate Research Programme’s CMIP5^47^ are used in this study: CCSM4, CESM1-CAM5, CMCC-CM, GFDL-CM3, MIROC5, MPI-ESM-LR, NorESM1-M. These models have captured the relationship between monsoon diabatic heating and descending motion in the Mediterranean^13^. Results from other CMIP5 model are comparable to those of the seven models listed above (not shown). All datasets provide monthly mean data with a 2.5° × 2.5° longitude-latitude grid at 17 vertical pressure levels for the variables. The model output can be obtained from the CMIP5 archive website, http://www.ipcc-data.org/sim/gcm_monthly/AR5/Reference-Archive.html. The analyses are performed using the ensemble mean of the seven CMIP5 models for JJA. The present (future) climate is the 20 years of the historical (RCP8.5) simulation from 1986 to 2005 (from 2081 to 2100) and is referred to as 20C (21C). The future change is also denoted by 21C minus 20C.

### Model configuration and experiment design

The linearized primitive equations based on the Linear Baroclinic Model (LBM) are used to diagnose the steady-state atmospheric dynamical response to prescribed diabatic heating^48^. The model has a horizontal resolution of T42 and 20 vertical levels in sigma coordinates (available online at http://ccsr.aori.u-tokyo.ac.jp/lbm/sub/lbm.html). The 20C JJA mean state for the LBM is prescribed from the average of the seven-model simulations. To investigate physical mechanisms that cause the changes in the Mediterranean atmospheric circulation, the LBM experiments are performed using the heating profile for a different region. The heating is estimated^49^ as a residual of the thermodynamic equation for the average of the seven-models. The four potential heating or cooling profiles are the South Asian summer monsoon (SASM; 75°–115° E, 0–40° N and 50°–75° E, 0–15° N), East Asian summer monsoon (EASM; 115°–180° E, 20°–50° N), Atlantic (AT; 45°–10° W, 10° S–10° N), and African (AF; 10° W–40° E, 0–15° N) regions (Supplementary Fig. S1 black boxes).

### Rossby wave theory and a ray tracing technique

The dispersion relationship for the barotropic Rossby wave on an horizontally nonuniform flow that considers a meridional wind components^50^ is expressed as $\omega ={\bar{u}}_{M}k+{\bar{v}}_{M}l+\frac{{\bar{q}}_{x}l-{\bar{q}}_{y}k}{{K}^{2}}$, where *ω* is frequency, $({\bar{u}}_{{\rm{M}}},\,{\bar{v}}_{{\rm{M}}})=(\bar{u},\,\bar{v})/cos\phi$ is the basic-state zonal and meridional flow, and the square of the total wavenumber is ${K}^{2}={k}^{2}+{l}^{2}$ with zonal and meridional wavenumbers of *k* and *l*, respectively. The zonal and meridional gradients of absolute vorticity ${\bar{q}}_{x}$ and ${\bar{q}}_{y}$ take the form ${\bar{q}}_{x}=\,\frac{1}{{a}^{2}cos\phi }(\frac{{\partial }^{2}\bar{v}}{\partial {\lambda }^{2}}-\frac{{\partial }^{2}\bar{u}}{\partial \phi \partial \lambda }cos\phi +\frac{\partial \bar{u}}{\partial \lambda }sin\phi )$ and ${\bar{q}}_{y}={\bar{\beta }}_{M}+\frac{{{\rm{\partial }}}^{2}\bar{v}}{{\rm{\partial }}\phi {\rm{\partial }}\lambda }+tan\phi \frac{{\rm{\partial }}\bar{v}}{{\rm{\partial }}\lambda }$, where ${\bar{\beta }}_{M}=\frac{{\rm{\partial }}f}{{\rm{\partial }}y}-\frac{{{\rm{\partial }}}^{2}{\bar{u}}_{M}}{{\rm{\partial }}{y}^{2}}$. The above dispersion relation provides the meridional wavenumbers for a specified zonal wavenumber $k$ for stationary waves ($\omega =0$); then the group velocities for the zonal and meridional directions can be estimated using ${C}_{gx}=\frac{{\rm{\partial }}\omega }{{\rm{\partial }}k}={\bar{u}}_{M}+\frac{({k}^{2}-{l}^{2}){\bar{q}}_{y}-2kl{\bar{q}}_{x}}{{K}^{4}}$ and ${C}_{gy}=\frac{{\rm{\partial }}\omega }{{\rm{\partial }}l}={\bar{v}}_{M}+\frac{({k}^{2}-{l}^{2}){\bar{q}}_{x}+2kl{\bar{q}}_{y}}{{K}^{4}}$. These group velocities can be converted to the ray of the wave activity by solving the following simple relations: $\frac{dx}{\partial t}={C}_{gx}$, and $\frac{dy}{\partial t}={C}_{gy}$. The location of the ray is calculated using the fourth-order Runge–Kutta method^51,52^.

### Classification of the sub-regions for the Mediterranean

The Mediterranean is divided into two regions separated at 20° E according to the future changes in the vertical motion^30^ (Fig. 1a shading); 1) Western Mediterranean (WM, including the Adriatic, Balearic, Ligurian, Tyrrhenian, and the western part of the Ionian Sea), and 2) Eastern Mediterranean (EM, including the Aegean, the eastern part of the Ionian, and the Levantine Sea).

### Statistical significance

The statistical significance of the multi-model ensemble mean in the change is determined by the criterion where the sign of anomalies agree that of the mean in more than 85% of models (Fig. 1 and Supplementary Figs S1 and S4).

### Observation and Reanalysis datasets

The monthly mean datasets for the period from 1979 to 2017 are used: (1) National Oceanic and Atmospheric Administration Extended reconstruction SST version 5 (ERSSTv5)^53^ dataset, and (2) European Centre for Medium-Range Weather Forecasts Reanalysis Interim (ERA-Interim)^54^ in the 500-hPa omega. The chlorophyll-a concentration is obtained from the Moderate Resolution Imaging Spectroradiometer (MODIS)^55^, covering the years from 2003 to 2017. For ease of comparison, all datasets are interpolated in to a 2.0° × 2.0° longitude-latitude grid. The fisheries landings datasets of two small pelagic species from 1985 to 2015 on the Food and Agriculture Organization’s (FAO) General Fisheries Commission for the Mediterranean (GFCM) are used: *Engraulis encrasicolus* (European anchovy) and *Sardina pilchardus* (European pilchard) are obtained from http://www.fao.org/gfcm/data/captur e-production. These two species are used because they were the most caught fish species in the Mediterranean.

## Supplementary information

Supplementary Information
