# Supplementary material for: Climate change over the Mediterranean and current destruction of marine ecosystem
Source: Sci Rep. 2019 Dec 11;9:18813. doi: 10.1038/s41598-019-55303-7 (PMC6906505; doi:10.1038/s41598-019-55303-7)
Supplement: Supplementary file 1 — Supplementary Information [file 41598_2019_55303_MOESM1_ESM.docx]

**Supplementary Information**

**Figure S1.** **Multi-model ensemble-mean of the vertically (1000**–**100hPa) averaged diabatic heating (Q1) for summer and future change**. Spatial pattern of Q1 (**a**) climatology (shading; K day^-1^) and (**b**) future change (shading; K day^-1^) for the seven-model ensemble-mean in boreal summer. Black dots indicate the regions where more than 85% of models agree on the sign of the mean for future change. Black boxes are the South Asian summer monsoon (SASM; 75°–115° E, 0–40° N and 50°–75° E, 0–15° N), East Asian summer monsoon (EASM; 115°–180° E, 20°–50° N), Atlantic (AT; 45°–10° W, 10° S–10° N), and African (AF; 10° W–40° E, 0–15° N) forcing areas. In (**a**,**b**) maps were generated by GrADS version 1.9b4 (http://grads.iges.org/grads/).

Figure S2. Vertical forcing profile. Vertical profiles of diabatic heating or cooling change (K day^-1^) averaged over the SASM (blue line), EASM (red line), Atlantic (green line), and African (black line) regions*.*

**Figure S3.** **Multi-model ensemble-mean summer basic states associated with the Rossby wave theory.** Climatological summer (**a**) 200-hPa zonal wind $\bar{U}$ (m s^-1^), (**b**) 200-hPa meridional wind $\bar{V}$ (m s^-1^), and (**c**) meridional gradient of the absolute vorticity $q_{y}$ (1.0🞨10^-11^ m^-1^ s^-1^) in the Mercator coordinate. Thick black contours represent zero $\bar{U}$ at 200 hPa. Maps were generated using GrADS version 1.9b4 (http://grads.iges.org/grads/).

**Figure S4. Multi-model ensemble-mean summer SST.** (**a**) 20C (1985–2005) mean summer SST (K) from the seven-model ensemble-mean in the CMIP5 historical scenario. (**b**) The same as in (**a**) but for future change (K) from the seven-model ensemble-mean in the CMIP5 historical and RCP8.5 scenarios. Dotted area in (**b**) indicates the region where more than 85% of model agree on the sign of the mean for future change. In (**a**,**b**) maps were generated by GrADS version 1.9b4 (http://grads.iges.org/grads/).


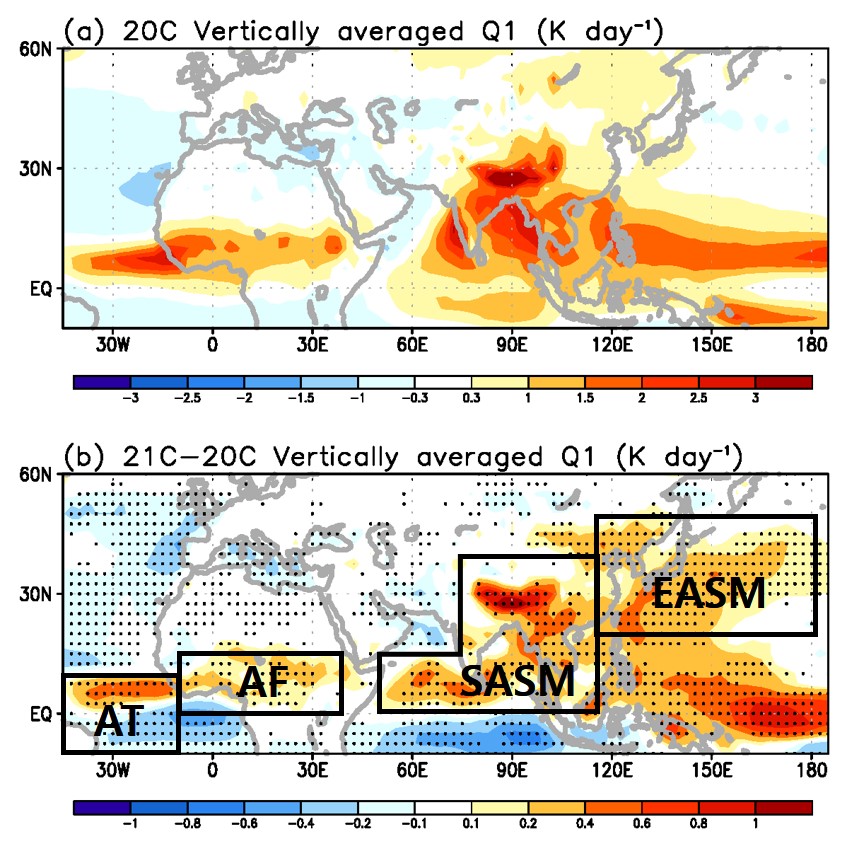


**Figure S1.** **Multi-model ensemble-mean of the vertically (1000**–**100hPa) averaged diabatic heating (Q1) for summer and future change**. Spatial pattern of Q1 (**a**) climatology (shading; K day^-1^) and (**b**) future change (shading; K day^-1^) for the seven-model ensemble-mean in boreal summer. Black dots indicate the regions where more than 85% of models agree on the sign of the mean for future change. Black boxes are the South Asian summer monsoon (SASM; 75°–115° E, 0–40° N and 50°–75° E, 0–15° N), East Asian summer monsoon (EASM; 115°–180° E, 20°–50° N), Atlantic (AT; 45°–10° W, 10° S–10° N), and African (AF; 10° W–40° E, 0–15° N) forcing areas. In (**a**,**b**) maps were generated by GrADS version 1.9b4 (http://grads.iges.org/grads/).


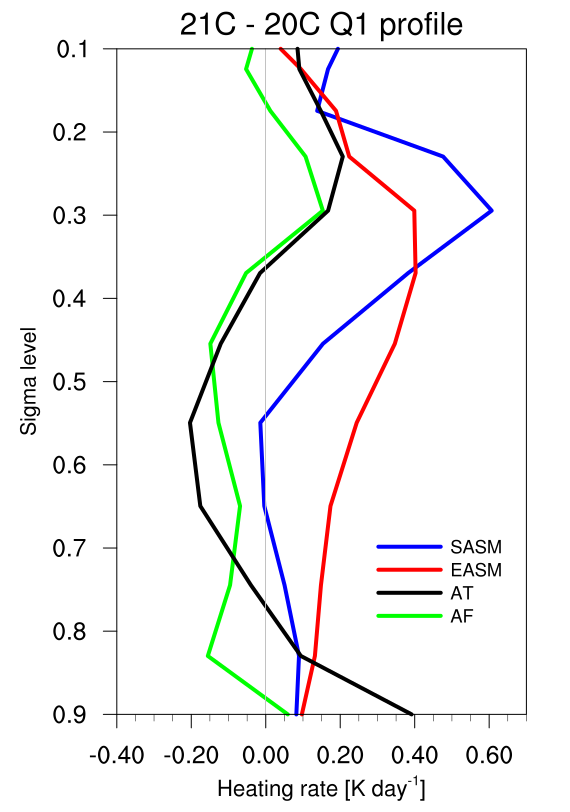


Figure S2. Vertical forcing profile. Vertical profiles of diabatic heating or cooling change (K day^-1^) averaged over the SASM (blue line), EASM (red line), Atlantic (green line), and African (black line) regions.


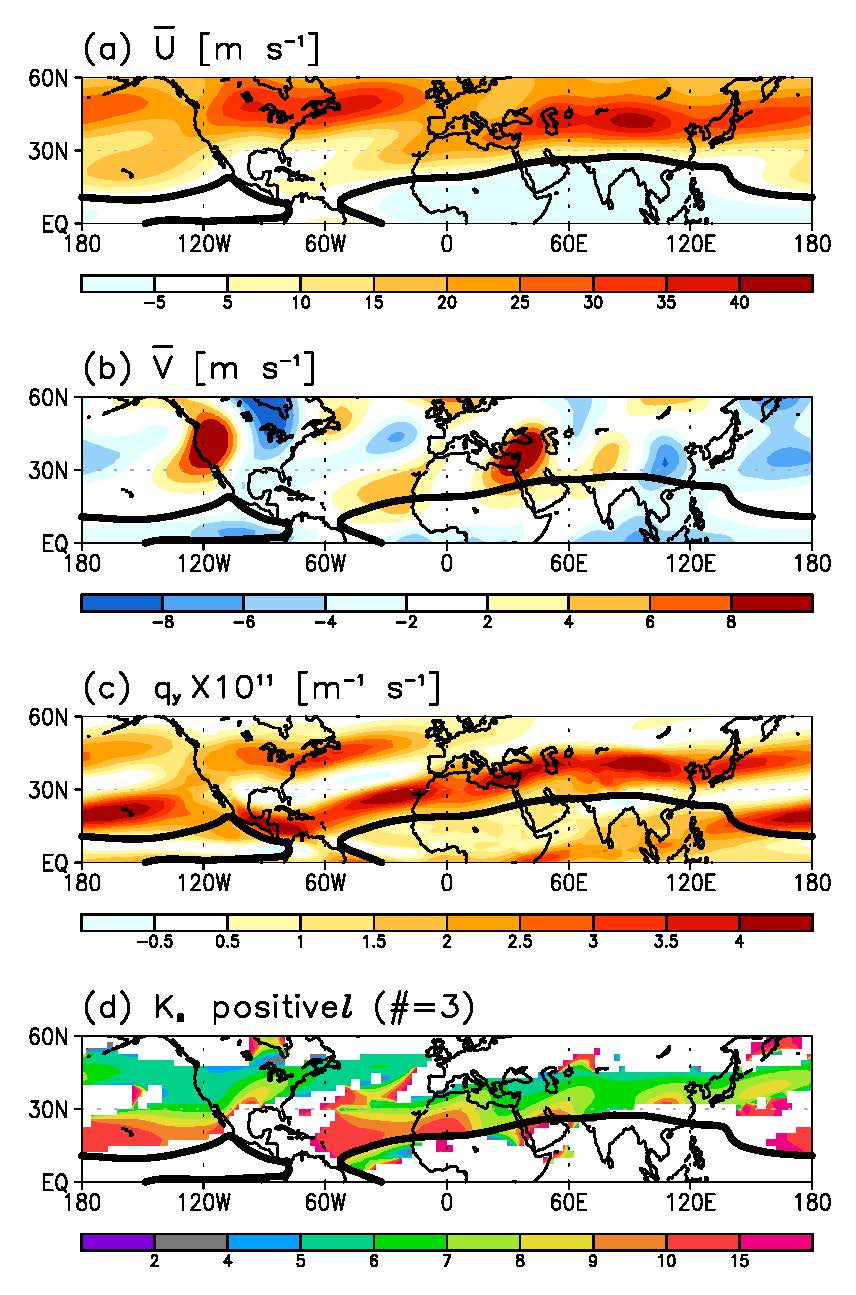


**Figure S3.** **Multi-model ensemble-mean summer basic states associated with the Rossby wave theory.** Climatological summer (**a**) 200-hPa zonal wind $\bar{U}$ (m s^-1^), (**b**) 200-hPa meridional wind $\bar{V}$ (m s^-1^), and (**c**) meridional gradient of the absolute vorticity $q_{y}$ (1.0🞨10^-11^ m^-1^ s^-1^) in the Mercator coordinate. Thick black contours represent zero $\bar{U}$ at 200 hPa. Maps were generated using GrADS version 1.9b4 (http://grads.iges.org/grads/).


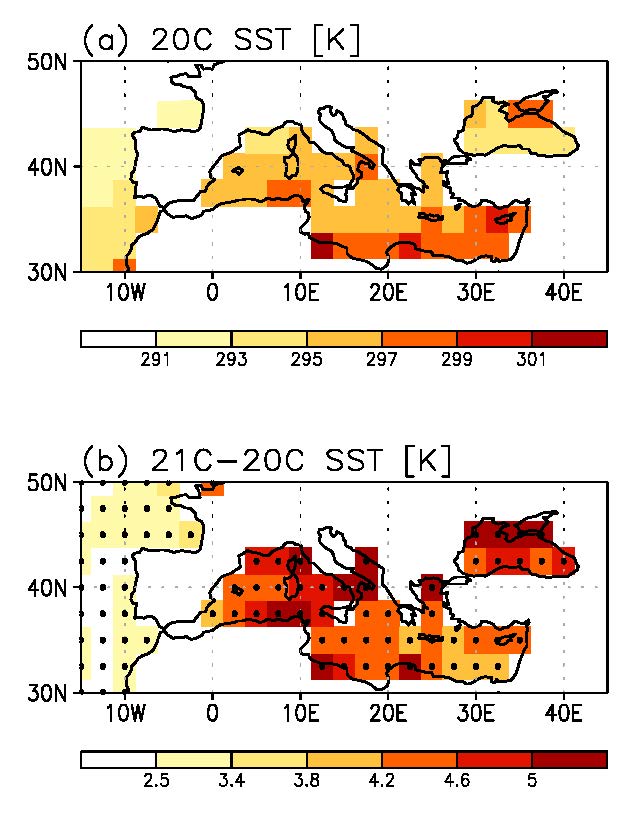


**Figure S4. Multi-model ensemble-mean summer SST.** (**a**) 20C (1985–2005) mean summer SST (K) from the seven-model ensemble-mean in the CMIP5 historical scenario. (**b**) The same as in (**a**) but for future change (K) from the seven-model ensemble-mean in the CMIP5 historical and RCP8.5 scenarios. Dotted area in (**b**) indicates the region where more than 85% of model agree on the sign of the mean for future change. In (**a**,**b**) maps were generated by GrADS version 1.9b4 (http://grads.iges.org/grads/).
